# Sulfasalazine-induced DRESS syndrome (Drug Rash with Eosinophilia and Systemic Symptoms)

**DOI:** 10.1590/S1516-31802008000400006

**Published:** 2008-07-03

**Authors:** Renata Telles Rudge de Aquino, Carmen Silvia Vieitas Vergueiro, Maria Elisa Ruffolo Magliari, Thais Helena Proença de Freitas

**Keywords:** Drug eruptions, Sulfasalazine, Hypersensitivity, Eosinophilia, Sulfonamides, Erupção por droga, Sulfassalazina, Hipersensibilidade, Eosinofilia, Sulfonamidas

## Abstract

**CONTEXT:**

DRESS syndrome (Drug Rash with Eosinophilia and Systemic Symptoms) is a type of drug reaction commonly mistaken for a viral infection. It must be recognized promptly due to its high morbidity and 10% mortality rate. Few cases of DRESS syndrome induced by sulfasalazine have been reported in the literature.

**CASE REPORT:**

The case of a 47-year-old white Brazilian woman who developed DRESS syndrome eight weeks after starting a course of sulfasalazine for treatment of seronegative arthritis is reported. She presented a skin rash, fever, hepatitis, lymphadenopathy, eosinophilia and atypical lymphocytes. The causative drug was discontinued immediately, but she only improved after treatment with prednisone.

## RESUMO

**CONTEXTO:**

A síndrome DRESS (*Drug Rash with Eosinophilia and Systemic Symptoms*) é um tipo de reação a drogas comumente confundida com uma infecção viral. Deve ser prontamente reconhecida devido a sua alta morbidade e taxa de mortalidade de 10%. Poucos casos de síndrome DRESS induzida por sulfasalazina foram relatados na literatura.

**RELATO DE CASO:**

É relatado o caso de uma mulher branca, brasileira, de 47 anos, que desenvolveu a síndrome DRESS oito semanas após iniciar tratamento com sulfasalazina para artrite soronegativa. A paciente apresentou *rash* maculopapular, febre, hepatite, linfadenomegalia, eosinofilia e linfocitose atípica. A medicação foi suspensa imediatamente no início dos sintomas, mas só houve melhora clínica após tratamento com prednisona.

## INTRODUCTION

With improvements in living conditions and increases in life expectancy, pharmaceutical drugs have become widely consumed. Healthcare professionals and consumers need to be aware of the vast range of adverse drug reactions, which are sometimes life-threatening.

In this report, a case of sulfasalazine-induced DRESS syndrome (the acronym for Drug Rash with Eosinophilia and Systemic Symptoms) is described. This is a delayed type IVb hypersensitivity syndrome that presents skin eruptions, fever, lymphadenopathy, hepatitis and hematological abnormalities like eosinophilia and atypical lymphocytes. It may also involve other organs. Its multi-organ involvement and frequent eosinophilia differentiates this entity from other common drug reactions.^1,2^

## CASE REPORT

A 47-year-old white Brazilian woman presented a sudden onset of fever of 38-39 ºC, chills, diffuse myalgia and severe asthenia. She had been taking sulfasalazine 500 mg daily for eight weeks for treatment of seronegative arthritis. She was not taking any other medications. The sulfasalazine was immediately discontinued. Despite the withdrawal of the drug, cervical and occipital lymphadenopathy, diffuse maculopapular rash and persistent fever were observed during the following week.

The laboratory tests showed: C-reactive protein 50 mg/l (normal range, NR < 5), sedimentation rate 9 mm (NR < 12), alanine amino transferase (ALT) 42 U/l (NR 7-35), aspartate amino transferase (AST) 34 U/l (NR 13-35), gamma glutamyl transferase (GGT) 35 IU/l (NR 11-50) and normal prothrombin time. The initial full blood count and urine test were normal. By the seventh day, she started to complain of abdominal pain, nausea, anorexia and occipital headache. Her clinical examination revealed painful enlargement of the liver. New tests, in a different laboratory with different methods of analysis, showed: C-reactive protein 4.77 mg/dl (NR < 0.50), sedimentation rate 15 mm (NR < 10), normal bilirubin level and elevation of serum amino transferase levels: ALT 387 U/l (NR 7-31 U/l), AST 217 U/l (NR < 31) and GGT 621 U/l (NR 8-41). The total white cell count was 16,980/mm^3^ (NR 3,500-10,500), eosinophil count 1,430/mm^3^ (NR 50-500), monocyte count 1,940/mm^3^ (NR 300-900), lymphocyte count 6,880/mm^3^ (NR 900-2,900), neutrophil count 6,555/mm^3^ (NR 1,700-8,000) and platelet count 259,000/mm^3^ (150,000-450,000). The peripheral blood contained few atypical lymphocytes but marked eosinophilia. Serological tests for the following infectious agents were negative: toxoplasmosis, dengue, mycoplasma, Epstein-Barr virus, Echovirus, Coxsackie, leptospirosis and hepatitis A, B and C. Tests for autoimmune antibodies (antinuclear and rheumatoid factor) were also negative.

It was concluded that the diagnosis was hypersensitivity to sulfasalazine. Prednisone was started at a dose of 40 mg/day. After one day, the patient felt much better and, after three weeks, her laboratory test results were normal, except for the gamma glutamyl transferase, which only went down to 148 IU/l (NR 11-50) but three weeks latter was normal. The prednisone dose was then reduced at a rate of 5 mg per week, and the patient continued to feel well.

## DISCUSSION

The acronym DRESS, which stands for Drug Rash with Eosinophilia and Systemic Symptoms, has been proposed as more specific than “hypersensitivity”, which would be an appropriate description for most types of drug reactions. DRESS denotes two important characteristics: multisystemic involvement and frequent eosinophilia.^1,2^ It has been estimated to occur in about one in 10,000 cases of exposure to drugs like antiepileptics and sulfonamides.^3^

The clinical manifestations typically occur within two to six weeks after starting the therapy and in most cases they cease when the drug is discontinued, without sequelae. However, a fatal outcome has been reported in 10% of such cases. The outcome from this syndrome is determined by the occurrence of visceral complications such as fulminant hepatitis, interstitial nephropathy, eosinophilic interstitial pneumopathy, pericarditis, myocarditis or pancreatitis.^4^

The differential diagnosis includes acute viral infections, idiopathic hypereosinophilic syndrome and lymphoma^1,2^

The pathophysiology of DRESS syndrome remains unclear. The prevailing hypothesis holds that the causative drug induces hypersensitivity as a result of abnormalities in the production and detoxification of its active metabolites. These abnormalities may be related to genetic predisposition, as the risk is increased in patients with a positive family history for DRESS syndrome, in individuals who are slow acetylators and in black patients. This hypothesis is now accepted for sulfonamides and anticonvulsants.^5^ In the case presented here, the patient's mother had been diagnosed with leukoclastic vasculitis, with marked eosinophilia while taking sulfasalazine. Special attention should be paid to herpes virus 6 (HHV6), since several papers have suggested a possible interaction between DRESS and reactivation or primary infection with HHV6.^5^

Aromatic antiepileptic agents (phenobarbital, carbamazepine or phenytoin), minocycline and allopurinol are the most common causes of DRESS. Sulfonamides, gold salts, cyclosporine, hydrochlorothiazide, D-penicillinamine, dapsone and imatinib may also induce DRESS syndrome.^3,5^ The treatment is not standardized at present. The suspected drug should be discontinued immediately. Delaying this measure may be associated with worse prognosis. Glucocorticoids remain the most widely used agents, although the dosages vary widely across case reports. Favorable results have been reported with its use,^1,5^ and in one case it was maintained as long-term treatment for the patient's joint disease.^5^ Several weeks or months are needed to determine whether recovery has been achieved. Recurrences have been reported, most notably in cases relating to anticonvulsant drugs.^5^

## CONCLUSION

Drug reactions must be considered by physicians in many different clinical settings. Recognition of DRESS syndrome, which may be mistaken for viral diseases, is of great importance because of its high morbidity and the need to immediately discontinue the use of the causative drug.
